# Determination of *Elizabethkingia* Diversity by MALDI-TOF Mass Spectrometry and Whole-Genome Sequencing

**DOI:** 10.3201/eid2302.161321

**Published:** 2017-02

**Authors:** Helle Brander Eriksen, Heidi Gumpert, Cecilie Haase Faurholt, Henrik Westh

**Affiliations:** Copenhagen University Hospital, Hvidovre, Denmark (H.B. Eriksen, H. Gumpert, H. Westh);; Copenhagen University Hospital, Frederiksberg, Denmark (C.H. Faurholt);; University of Copenhagen, Copenhagen, Denmark (H. Westh)

**Keywords:** *Elizabethkingia* species, *Elizabethkingia miricola*, nosocomial infections, multidrug resistance, carbapenemase, β-lactamase, GOB-1, 16S rRNA, septic arthritis, Etest, antimicrobial resistance, bacteria, MALDI-TOF, matrix-assisted laser desorption/ionization time-of-flight mass spectrometry, nomenclature

## Abstract

In a hospital-acquired infection with multidrug-resistant *Elizabethkingia*, matrix-assisted laser desorption/ionization time-of-flight mass spectrometry and 16S rRNA gene analysis identified the pathogen as *Elizabethkingia miricola*. Whole-genome sequencing, genus-level core genome analysis, and in silico DNA-DNA hybridization of 35 *Elizabethkingia* strains indicated that the species taxonomy should be further explored.

*Elizabethkingia* are gram-negative, nonmotile, nonspore-forming rods that are rarely associated with human disease. *E. meningoseptica* is the most recognized clinically, mainly causing hospital-acquired infections, including sepsis in immunocompromised patients and meningitis in neonates (*1*). These bacteria are intrinsically resistant to β-lactam antimicrobial drugs, including second- and third-generation cephalosporins and carbapenems, because they naturally carry an Ambler class A serine β-lactamase gene *bla*_CME_, which encodes a penicillin- and cephalosporin-hydrolyzing enzyme (*2*), and 2 Ambler class B metallo-β-lactamase genes, *bla*_B_ and *bla*_GOB_, which encode carbapenem-hydrolyzing enzymes (*3*). 

The species *E. anopheles* and *E*. *miricola* are increasingly being isolated and cause similar infections to *E. meningoseptica* (*4*,*5*). *E*. *miricola* was discovered in condensed water collected onboard the Russian space station Mir in 2003 (*6*), and *E. anopheles* was isolated from the midgut of the *Anopheles gambiae* mosquito in 2011 (*7*). We report infection with a carbapenem-resistant *Elizabethkingia* species in a patient in Copenhagen, Denmark in September 2015; we identified the bacterium as *E. miricola* by using MALDI-TOF (matrix-assisted laser desorption/ionization time-of-flight) mass spectrometry. We further studied the isolate using whole-genome sequencing (WGS) and compared it with other available *Elizabethkingia* genomes.

## The Study

The patient was a 64-year-old man with recurrent erysipelas of both legs. He was initially hospitalized with erysipelas in August 2015 and successfully treated with penicillin and dicloxacillin. On readmission in September 2015, he was again treated with intravenous penicillin and dicloxacillin. Six days later, the patient felt increased pain, and his knee became warm and sore. These findings were interpreted to be symptoms of reactive arthritis caused by a urinary tract infection, and his drug treatment was changed to cefuroxime. The following day, a cloudy synovial fluid was aspired from his knee, so ciprofloxacin was added to his drug regimen. Because of aggravating symptoms and increasing C-reactive protein levels, the patient’s treatment was then changed to piperacillin with tazobactam, after which his symptoms improved. The patient was discharged with oral dicloxacillin and ciprofloxacin, and 4 weeks later he was well.

An overnight culture of the synovial fluid from the patient’s knee grew multidrug-resistant, gram-negative, nonmotile rods. MALDI-TOF (MALDI Biotyper 3.1, Bruker Daltonics Microflex LT, database MBT DB-5627) mass spectrometry identified the colonies as *E. miricola* (score 2.105). Antimicrobial susceptibility testing showed that the isolate was susceptible to ciprofloxacin (MIC 0.25 mg/L) and co-trimoxazole (MIC 0.125 mg/L); intermediately susceptible to piperacillin/tazobactam (MIC 16 mg/L), amoxicillin/clavulanic acid (MIC 8 mg/L), and tigecycline (MIC 0.5 mg/L); and resistant to all other drugs tested (Table). The imipenem/imipenem-EDTA Etest (bioMérieux, Marcy l’Etoile, France) for detection of metallo-β-lactamase was positive.

**Table:** Antimicrobial susceptibility of *Elizabethkingia* isolate HvH-WGS333 from patient with hospital-acquired septic arthritis, Copenhagen, Denmark 2015*

Antimicrobial	MIC, mg/L	MIC breakpoint, S≤/R>, mg/L	Interpretation
Ciprofloxacin	0.25	0.5/1	Susceptible
Co-trimoxazole	0.125	4/4	Susceptible
Tigecycline	0.5	0.25/0.5	Intermediate
Piperacillin/tazobactam†	16	4/16	Intermediate
Amoxicillin/clavulanic acid‡	8	2/8	Intermediate
Ampicillin	>256	2/8	Resistant
Cefuroxime	>256	4/8	Resistant
Ceftazidime	>256	4/8	Resistant
Meropenem	>32	2/8	Resistant
Gentamicin	12	4/4	Resistant
Colistin	>256	4/4	Resistant

*Submitted isolate to GenBank (accession no. NZ_MCJF00000000.1). The isolate contains antimicrobial resistance genes *bla*_B-4_, *bla*_GOB-13_, and an extended-spectrum β-lactamase, sharing 90.6% nucleotide identity to *bla*_CME-1_. Antimicrobial susceptibility testing was performed by using Etest (bioMérieux, Marcy l’Etoile, France) according to European Committee on Antimicrobial Susceptibility Testing (EUCAST) guidelines (http://www.eucast.org). For interpretation, non–species-related (pharmacokinetics/pharmacodynamics) breakpoints were used except for co-trimoxazole, in which *Stenotrophomonas malthophilia* breakpoints were used, and for gentamicin and colistin, in which *Pseudomonas* sp. breakpoints were used. R, resistance; S, susceptibility. †For susceptibility testing purposes, the concentration of tazobactam was fixed at 4 mg/L.  ‡For susceptibility testing purposes, the concentration of clavulanic acid was fixed at 2 mg/L.

To confirm the species identity and identify the antimicrobial resistance genes, we performed WGS of the isolate with the Illumina MiSeq platform producing 2 × 150-bp paired-end reads by using the Nextera XT library preparation kit (Illumina Denmark ApS, Copenhagen, Denmark). Reads were assembled by using SPAdes, which produced a 4.13-Mbp genome termed HvH-WGS333 (BioProject PRJNA335686, BioSample SAMN05450241). We performed annotation of the genome using Prokka (*8*) and used ARG-ANNOT (*9*) to identify 2 metallo-β-lactamase genes, namely *bla*_B-4_ and *bla*_GOB-13_, and an extended-spectrum β-lactamase gene sharing 90.6% nucleotide identity with *bla*_CME-1_. The isolate was identified by EzTaxon (*10*) as *E. miricola* on the basis of its 16S rRNA gene with 99.69% similarity. However, single-nucleotide variant (SNV) analysis by read alignment with Bowtie2 and SNV-calling with SAMtools comparing HvH-WGS333 and the GTC 862 *E. miricola* strain (accession no. NZ_LSGQ00000000) yielded ≈78,000 SNVs. Furthermore, analyzing the coverage breadth by using BEDTools revealed that this isolate shared 80.5% of this *E. miricola* genome.

Because the isolate from this patient was not closely related to the *E. miricola* type strain, we performed a genus-level core genome analysis of 35 publicly deposited *Elizabethkingia* strains from GenBank to identify a more closely related isolate. Each genome was annotated (*8*) and the core genome was approximated by ROARY (*11*), yielding a 419,813-bp core genome of 426 genes. We constructed a phylogenetic tree of the core genome using RAxML (*12*) with 100 bootstrapping replicates (Figure). The ATCC 13253 *E. meningoseptica* strain did not cluster with the other *E. meningoseptica* strains and was the most distantly related *Elizabethkingia* sp. analyzed. Before WGS was commonly used, high levels of divergence were reported among *E. meningoseptica* (formerly *Flavobacterium meningosepticum*) isolates, with 2 groups detected, Ursing and Bruun (UB) I and UBII (*13*), with ATCC 13253 belonging to UBI. Our HvH-WGS333 isolate was most closely related to EM_CHUV, an endotracheal isolate submitted as *E. miricola*; these 2 isolates are next most closely related to G4071, which belongs to the UBII:2 (*13*), and then to GTC 862 *E. miricola*. *E. miricola* ATCC 33958 is most closely related to G4075, a clinical blood culture *E. meningoseptica* in the UBII:3 group. Strains belonging to groups UBI and UBII are phenotypically very similar, explaining why DNA-DNA hybridization (DDH) was previously used to distinguish these groups (*14*).

**Figure:**
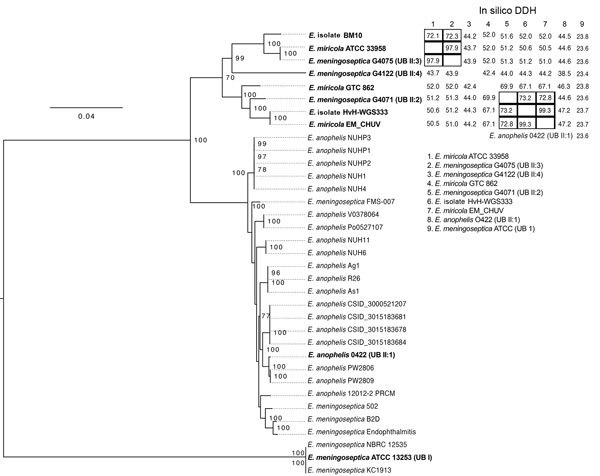
Phylogenetic tree of *Elizabethkingia* isolate from a patient with hospital-acquired septic arthritis in Copenhagen, Denmark 2015 compared with reference strains and in silico DNA-DNA hybridization (DDH). Tree was produced by using the *Elizabethkingia* core genome from all publicly available *Elizabethkingia*. Bootstrapping support was implemented by running 100 replicates, with values >70% indicated on branches. Initial species identification followed by NCBI isolate name is indicated in the tree. Isolates assigned into Ursing and Bruun (UB) groups and subgroups are indicated in brackets following the isolate name. Table at top right indicates the in silico DDH values; black boxes indicate isolates with in silico DDH >70% indicating they belong to the same species.​ The *E. meningoseptica* isolates NBRC 12535, ATCC13253, and KC1913 seem to be identical strains. Scale bar indicates nucleotide substitutions per site.

Additionally, we performed in silico DDH (*15*) to other sequenced *Elizabethkingia* isolates to determine whether our isolate could be defined as the same species (i.e., having a DDH value >70%). The in silico DDH value is calculated by summing up the identities in matches between the genome sequences and dividing by the total match hit lengths. HvH-WGS333 only had an in silico DDH >70% with EM_CHUV and G4071, which clustered most closely together in the tree but was also closely related (67.1%) to strain GTC 862 (Figure). A previous DDH study investigating the *Elizabethkingia* genus found DDH values at 70°C of 52% between UBII:2 and UBII:3, 45% between UBII:2 and UBII:4, and 36% between UBII:3 and UBII:4 (*14*), in agreement with our in silico DDH results (Figure). Our analyses support the identification of the 3 UBII subgroups 2, 3, and 4 as being 3 different *Elizabethkingia* species, with UBII:2 being the closest related to the *E. miricola* type strain.

## Conclusions

The wide adoption of MALDI-TOF mass spectrometry for bacterial identification is likely to increase the detection of infections caused by *Elizabethkingia*, and thus the number of infection reports. However, the applied MALDI-TOF database contained only 3 *E*. *meningoseptica* and 1 *E*. *miricola* isolates at the time of our study. Identification on the basis of the 16S rRNA gene corroborated our MALDI-TOF mass spectrometry identification of *E*. *miricola.* When exploring this further using WGS, we discovered our isolate shared 80.5% of the GTC 862 *E. miricola* genome and the in silico DDH value was 67.1%, just below the 70% species threshold. It thus appears that our isolate, along with EM_CHUV and G4071, is closely related to the *E. miricola* species and that the taxonomic position of the UBII:3 and UBI:4 strains should be reconsidered. In light of our WGS analysis demonstrating considerable *Elizabethkingia* genetic diversity, we suggest that the MALDI-TOF database needs to be updated and the nomenclature needs to be re-examined. WGS is proving to be a valuable tool for the correct identification of new species and detailed characterization of multidrug-resistant bacteria.
